# Physiological impact of amino acids during heat stress in ruminants

**DOI:** 10.1093/af/vfad052

**Published:** 2023-10-13

**Authors:** Juan J Loor, Vincenzo Lopreiato, Valentino Palombo, Mariasilvia D’Andrea

**Affiliations:** Department of Animal Sciences, Division of Nutritional Sciences, University of Illinois, Urbana, IL 61801, USA; Department of Veterinary Sciences, Università degli Studi di Messina, Viale Palatucci snc 98168, Messina, Italy; Department of Agricultural, Environmental and Food Sciences, Università degli Studi del Molise, via De Sanctis snc 86100, Campobasso, Italy; Department of Agricultural, Environmental and Food Sciences, Università degli Studi del Molise, via De Sanctis snc 86100, Campobasso, Italy

**Keywords:** lactation, metabolism, nutrigenomics

**Implications**

- High seasonal heat and humidity decrease postruminal nutrient supply, deteriorate the ruminal and intestinal mucosa, and facilitate translocation of bioactive molecules into the bloodstream.
- Inflammation, oxidative stress, and misfolding of cellular proteins are physiological hallmarks of stress, including heat and humidity. These processes divert amino acid use away from productive purposes.
- Heat-stressed ruminants could benefit from increases in postruminal supply of arginine, cysteine, leucine, lysine, and methionine.
- One-carbon metabolism generates antioxidants from several indispensable and nondispensable amino acids as well as folic acid, choline, and betaine.
- ‘Omics’ tools are allowing discovery of physiological mechanisms that can be manipulated via the supply of specific amino acids to alleviate the impact of heat stress.

## Introduction

### Heat stress and ruminant production

The loss in value due to reductions in milk and meat production from heat stress (HS) *worldwide* by the end of the century have recently been estimated at $14.9 to $39.9 billion per year (Thornton et al., 2021). These losses are predicted to be most-severe in tropical regions where an increase in beef production, for example, to feed the growing population worldwide is expected by 2050 (Cooke et al., 2020). Common HS abatement practices including increasing shaded areas, increasing air velocity by use of fans, and the use of water-soaker lines to increase evaporative heat loss have been implemented on farms. Despite this, the continued increase in environmental temperature and the duration and frequency of droughts will impact not only animal numbers, but also productive efficiency worldwide and the economic return (Wankar et al., 2021).

Although tackling the negative impact of global warming on productive efficiency and wellbeing requires multiple management approaches, greater knowledge of physiological mechanisms that are altered by HS will be valuable. This is particularly true in parts of the world where intensive production systems are characterized by animals of high-genetic merit that not only has resulted in marked increases in milk or beef production, but also in the amount of heat (i.e., heat increment) produced per unit of feed consumed (West, 2003; Cooke et al., 2020). Escalating global temperatures combined with global demand for livestock products has resulted in HS becoming an important ongoing challenge facing the global livestock industry.

## Physiological Outcomes During Heat Stress

### Major cellular events

Work in model organisms clearly established that the key cellular adaptation driven by short- or long-term exposure to temperatures above the upper range (i.e., HS) is the misfolding and aggregation of misfolded proteins leading to impaired cellular function and disorders, including endoplasmic reticulum (ER) stress (Tyedmers et al., 2010). The importance of these cellular events stems from the fact that functionality of most proteins in the cell requires the formation of a proper three-dimensional structure, a process termed “folding”. Studies in the last 20 years using transcriptome, proteome, metabolome, and/or systemic biomarker analyses have confirmed that these cellular events occur in experimentally or seasonal HS cattle, i.e., animals experiencing increases of body temperature (from 1- to 2-wk in experimental challenges to several wk in seasonal challenges) in the range of 0.2 to 1.5 °C (Bernabucci et al., 2002; Kim et al., 2018; Pate et al., 2020; Koch et al., 2021, 2023; Ruiz-Gonzalez et al., 2023).

Heat shock protein (HSP) HSP70 is a key “chaperone” in the protein quality control system that cells possess to facilitate the folding or refolding of misfolded proteins, hence, preventing protein aggregation. Upregulation of the spliced X-box binding protein 1 (XBP1s), a member of the cAMP-response element-binding/activating transcription factor b-ZIP family of transcription factors, is another key cellular response to HS that helps decrease protein misfolding (Loor and Elolimy, 2022). Production of HSP is controlled by heat shock factors (HSF, e.g., HSF1), which upon phosphorylation are activated and travel to the nucleus where they bind to heat shock elements and enhance transcription of HSP among other important targets (Tyedmers et al., 2010). The imbalance between reactive oxygen species (ROS) production and the available antioxidant defenses against them can induce oxidative stress, and is another key biological process that causes protein unfolding and misfolding (Tyedmers et al., 2010). Heat stress in livestock species including dairy cows and beef cattle is characterized by oxidative stress, inflammation, ER stress, and increased gut permeability (Cooke et al., 2020; Ma et al., 2021; Ruiz-Gonzalez et al., 2023) all of which can be assessed through tissue and systemic biomarkers such as inflammation-related proteins (e.g., cytokines, acute-phase proteins (APP), lipopolysaccharide binding protein). The sensitivity of these cellular systems to HS in ruminants is exemplified by the 5-fold increase in abundance of XBP1s in mammary tissue from cows experiencing subtle increases in body temperature (0.2–0.3 °C) (Pate et al., 2020, 2021).

### Ruminal and intestinal responses to heat stress

Although reductions in dry matter intake (DMI) as a result of HS are a well-known response in cattle and small ruminants (West, 2003; Cooke et al., 2020; Mishra, 2021), it is not often recognized (at least in dairy cows) that under those conditions the animal may increase the frequency of intake and water consumption as a “self-regulatory” mechanism with consequent impacts on ruminal fermentation, e.g., greater production of VFA and lower pH over extended periods of time (West, 2003). Such responses are expected to be more severe the longer that the animal experiences HS (Hou et al., 2021).

Two studies recently evaluated responses of the ruminal epithelium in HS versus pair-fed (thermoneutral) lactating cows during a chronic mild [temperature-humidity index (THI) = 76] (Eslamizad et al., 2020) or moderate (THI = 83) (Guo et al., 2021) HS period using environmental chambers. The “pair-fed” approach helps determine the direct effects of HS without the confounding effect of reduced DMI. Although the mild HS did not alter genes or proteins in ruminal epithelium associated with tight-junctions (Eslamizad et al., 2020), histological analysis under moderate HS revealed greater shedding of the stratum corneum (Guo et al., 2021). This change was associated with reduced pH and greater total VFA concentrations in the rumen, with little effect on lipopolysaccharide (LPS) concentrations [an inflammatory molecule; (Plaizier et al., 2018)]. Beyond LPS, it is likely that negative impacts of HS on the ruminal epithelium would enhance the transport of other bioactive molecules as has been reported during episodes of subacute ruminal acidosis (Plaizier et al., 2018).

The impacts of HS (THI = 76) on the small intestine (tissue and mesenteric lymph nodes) were reported recently in a study involving lactating cows exposed to HS for 4-d through heat chambers (Koch et al., 2019, 2021, 2023). Transcriptomics revealed a more predominant population of immune cells (mainly macrophages) in the jejunal epithelium as opposed to cells typical of intestinal epithelial lineage that were more predominant in pair-fed cows under thermoneutral conditions. Despite these differences, there was no impact of HS on the jejunal morphology, an effect that was evident in ruminal epithelium albeit when animals were exposed for a longer period (7-d) to moderate HS (THI = 83) (Guo et al., 2021). Other unique biological differences between ruminal and jejunal epithelium responses to HS pertain to molecular characteristics of the tissues. For example, whereas ruminal epithelium from cows experiencing HS exhibited a marked downregulation of genes associated with an inflammatory response, jejunal epithelium exhibited downregulation of some key tight junction proteins (tight junction protein 1, claudin 1 mRNA), upregulation of catalase, and upregulation of alkaline phosphatase (ALP). Some of these responses were also verified at the protein level, e.g., downregulation of catalase, and also highlighted upregulation in the abundance of several HSP (Koch et al., 2021). The decrease in abundance of tight-junction proteins has been associated with increased permeability and LPS transport in pigs, suggesting “gut leakiness” (Baumgard and Rhoads, 2013). Catalase responds to increases in hydrogen peroxide production (a non-radical ROS), and helps detoxify it to water. Activity of ALP helps bind LPS and limit inflammation in the gut lumen (Koch et al., 2019). Clearly, differences in the response of the ruminal and intestinal epithelium to HS might be partly due to the unique biological function of each tissue along with the characteristic differences of the luminal environment in these sections of the gut. However, what seems evident is that reductions in tissue permeability, especially during longer-term HS, would represent a “double whammy” of physiological stress for the animal.

**Figure 1.**
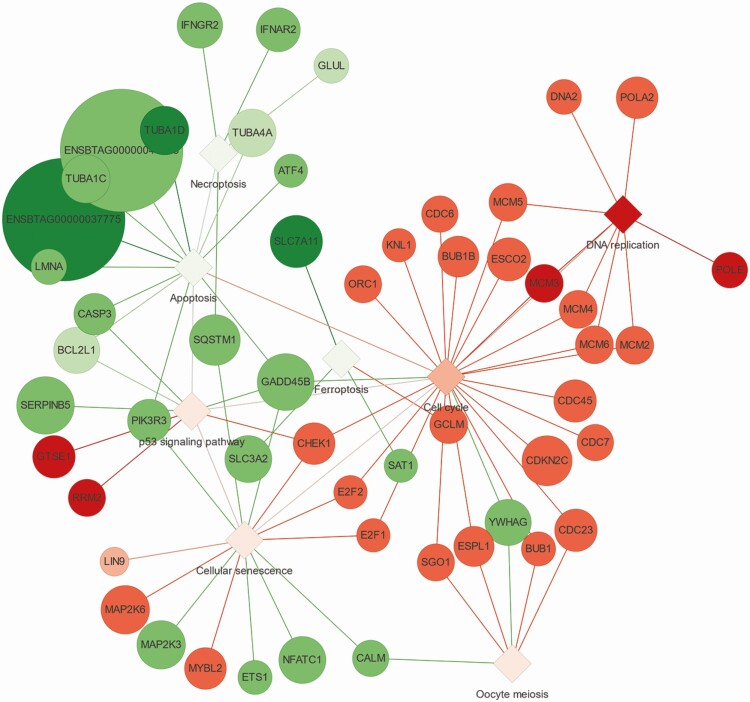
Gene networks associated with ‘Cell cycle’ and ‘DNA replication and repair’ in the Kyoto Encyclopedia of Genes and Genomes (KEGG) pathway database generated from differentially expressed genes using RNAsequencing data from ruminal epithelium of cows experiencing heat stress relative to pair-fed cows in thermoneutral conditions (Guo et al., 2021). Reanalysis of the data using R resulted in 350 differentially expressed genes. The PANEV tool in R (Palombo et al., 2020) was used to generate the visual representation of the pathways based on the differentially expressed gene list. The orange and red colors denote upregulation and shades of green downregulation of pathways and genes.

Transcriptomics data have revealed unique physiological responses of the ruminal epithelium to an extended period of HS. Of importance in the context of nutrition is the marked upregulation of genes encoding enzymes in metabolic pathways associated with amino acid (AA) metabolism, fatty acid degradation, and glycolysis (out of ~500 differentially expressed) (Guo et al., 2021). It was striking that among the most-affected and upregulated biological pathways were “DNA replication” and “Cell cycle” (Figure 1) (Guo et al., 2021). These molecular data were discussed in the context of histological and ruminal fermentation parameters, and authors proposed that during sustained HS restoring the normal epithelial tissue morphology takes precedent, but likely channels the use of nutrients such as AA, fatty acids, and butyrate towards energy production to sustain the increase in “repair” of the damaged epithelium (Guo et al., 2021). In fact, it is likely that this sort of repair responses at the ruminal and intestinal levels during HS are important determinants of the reduction in supply of energy-generating nutrients to the mammary gland that often leads to lower milk protein synthesis (Ma et al., 2019; Hou et al., 2021).

### Major systemic effects of heat stress on metabolic functions

Skeletal muscle in dairy and beef cattle seems to exhibit decreased insulin sensitivity and is more responsive to circulating cortisol during HS, events which are thought to promote the mobilization of AA (i.e., proteolysis) as gauged by changes in plasma AA and increased plasma urea nitrogen concentrations (Cowley et al., 2015; Gao et al., 2017; Kim et al., 2022). However, the fact that abundance of proteins associated with skeletal muscle proteolysis (e.g., Calpain I and II, E3 ligases) did not change in cows that experienced HS via heat blankets for 7 d (had a 1.3 °C increase in body temperature) suggested that protein breakdown might not be the cause for increases in plasma AA (Roths et al., 2023). Some of these physiologic changes partly explain the decrease in milk yield or daily weight gain, as the AA supply may be moved away from anabolic organs during times of HS (Baumgard and Rhoads, 2013). Thus, besides a shortage in postruminal AA supply for production purposes due to reduced DMI, HS can also hamper a successful immune and antioxidant adaptation to hyperthermal conditions. This scenario further emphasizes the concept that proper nutrition (i.e., supply of specific nutrients) could help animals during periods of stress, reducing adverse effects and enhancing recovery (Li et al., 2007; Iseri and Klasing, 2014).

The increase in insulin, a potent anabolic hormone, during HS is seemingly a biological paradox (Baumgard and Rhoads, 2013), and recent data from experimentally HS lactating cows suggested that alterations in calcium signaling contribute to this response (Ruiz-Gonzalez et al., 2023). In a recent study with late-lactation cows, mTOR and some insulin signaling-related proteins were upregulated in liver and mammary tissue of cows under HS relative to pair-fed animals (Coleman et al., 2022a, 2022b). The ruminant liver is primarily gluconeogenic and the abundance of gluconeogenic enzymes during HS has been reported to increase in multiple species, including dairy cattle (Baumgard and Rhoads, 2013). It is well-established in nonruminants that when stimulated by insulin, SLC2A4 translocates to the plasma membrane and mediates insulin-stimulated glucose uptake. Hence, the upregulation of INSR and SLC2A4 proteins in liver tissue from HS cows led to the suggestion that insulin signaling and glucose uptake were upregulated. Insulin binding to its receptor leads to phosphorylation and activation of AKT through the phosphoinositide 3-kinase signaling cascade. Thus, the activation of AKT in liver tissue from HS cows (Coleman et al., 2022a) provided further indication that insulin signaling in liver was upregulated during HS. Although AKT was not activated by HS in mammary tissue, likely because lactating bovine mammary is insulin-insensitive, both the activation of mTOR and a key protein responsible for translation (eukaryotic translation elongation factor 2) suggested channeling of AA away from milk protein synthesis and towards synthesis of HSP or other “protective” proteins (Coleman et al., 2022b). Although further research is needed, the increase in insulin signaling and glucose uptake assessed in the liver of HS cows might have been linked to a greater demand of energy substrates to maintain all the mechanisms required for thermic regulation.

In the context of the insulin response, it should be kept in mind that during HS the synthesis of HSP in various tissues and cell types is increased in beef (Kim et al., 2022) and dairy cattle (Koch et al., 2021; Ma et al., 2021). Furthermore, Coleman et al. (2022a) reported that the abundance of HSP70 in liver of HS cows increased approximately 150% relative to pair-fed cows. Hence, it is possible that insulin signaling was upregulated by HS in liver tissue to support increased AA uptake and utilization for HSP or APP synthesis. The latter hypothesis is supported by the observed increases in plasma concentrations of serum amyloid A (SAA), haptoglobin, and LPS-binding protein in HS cows (Pate et al., 2021; Ruiz-Gonzalez et al., 2023). Unlike mammary gland, such an effect of insulin on HSP and APP in the liver likely occurred independent of mTOR signaling, i.e., only a modest upregulation was detected for p-mTOR, the active form of this kinase.

### Amino acids and the response to stress

#### General mechanisms: nonruminants

It has been known for several decades that periods of physiological stress such as the transition into lactation, injury, trauma, HS, or infection lead to substantial mobilization of muscle protein (Li et al., 2007; Baumgard and Rhoads, 2013). Although precise mechanisms controlling the catabolic response of muscle in ruminants are not well-known, there is clear evidence that some of the AA liberated contribute to gluconeogenesis especially when the rate of DMI is low (Larsen and Kristensen, 2013). In nonruminants, increased protein mobilization during stressful conditions is induced by the actions of cytokines and stress hormones (e.g., glucocorticoids, glucagon, and epinephrine) (Chalvon-Demersay et al., 2021). Thus, it is believed that mobilization of body protein confers “adaptive advantage” by way of allowing the AA released to support defense mechanisms that are activated in the body. Although few data are available in ruminants, an example of this concept that is well-studied in nonruminant livestock is the “repartitioning” of AA use during pathogen challenges toward replenishing, for example, intestinal epithelial damage and also fuelling the immune system (Chalvon-Demersay et al., 2021). Even during stressful challenges, however, muscle-derived AA are not enough to reverse the negative impacts of stress, thus, increasing the usefulness of dietary supplementation of specific AA.

Utilization of AA to mount an immune response [e.g., acute-phase response (APR) or the adaptive response] can be itemized into “needs” associated with development of cells and molecules that optimize protection in the animal (e.g., APP), the needs to maintain this mature system, and the costs incurred by using the system to alleviate the stress (Iseri and Klasing, 2014). An example of the changing needs for AA during the APR was an initial estimation that lysine content in the systemic immune system of growing chicks increased from 1.8% of total body lysine before, to 10% post-LPS challenge (Iseri and Klasing, 2014). Subsequent studies determined more specifically the use of lysine during the APR and adaptive phases after an *E. coli* challenge. There was a marked increase in lysine content in both the circulating APP and liver tissue at 24 h post-*E. coli* followed by a marked decrease at 5 days postchallenge (Iseri and Klasing, 2014). The synthesis of APP was the biggest sink of lysine during the early immune response, while in the late-response antibody production and leukocytes also became important sinks for lysine. However, quantitatively, the accretion of lysine by the liver postchallenge was much greater than in cells or the APP, likely underscoring the central role of this organ in the production of the APP, e.g., haptoglobin, C-reactive protein and SAA as the major APP in ruminants (Pate et al., 2021; Ruiz-Gonzalez et al., 2023). Although not studied, it is likely that muscle protein mobilization provided other AA needed for APP synthesis as reported in sheep, e.g., phenylalanine and tryptophan (Hoskin et al., 2016; McNeil et al., 2016).

There are reported benefits in poultry and swine for increasing the supply of methionine, lysine, threonine, arginine, glutamine, aspartate, serine, tryptophan, glutamate and total sulphur AA on various measures of intestinal health and systemic antioxidant and antiinflamatory responses (Li et al., 2007). In those species, the impact of these AA can be classified in the context of how much of an effect they have for “supporting”, “supporting and restoring”, or just “restoring” physiological indicators (e.g., morphology, molecular) of optimal gut epithelial integrity and function, immune response, and antioxidant status. Benefits of specific AA stem from their known functions in a given biological process/es, e.g., glutamine and glutamate are readily used (instead of glucose) by the intestine to meet energy demands (Chalvon-Demersay et al., 2021). Lymphocyte proliferation is glutamine-dependent, and inflammatory challenges increase its utilization by lymphocytes (Li et al., 2007). In nonruminants under pro-inflammatory conditions driven by stressors, increased uptake of glutamine by the liver enhances glutamate synthesis and that of the antioxidant glutathione (GSH). Cysteine is also known to play a role in the regulation of lymphocyte function and as a precursor of GSH in nonruminants (Li et al., 2007). Overall, the metabolism of AA during stress conditions is complex, but substantial information for the unique role of a number of AA is available in nonruminants, including humans.

#### Ruminants

Unlike nonruminant livestock, much less is known on the ability of specific AA to help alleviate stress in ruminants arising from “normal” physiological events in the life of the animal such as the transition into lactation or from environmental challenges such as exposure to infections (viral or bacterial) or HS. Perhaps the most-studied AA in the context of stress are glutamine and glutamate (Li et al., 2007). Unlike non-ruminants, production of ATP by bovine lymphocytes from glutamine accounted for only 30% of the total, while most was metabolized to ammonia, glutamate, CO_2_, and aspartate (Li et al., 2007). Such a response is not surprising as glucose use in the ruminant, especially lactating animals, is channeled toward the mammary gland for milk synthesis (Larsen and Kristensen, 2013). The benefits of parenteral glutamine administration were evaluated in dairy cows from parturition to 7-d postpartum, a period where the animal experiences substantial metabolic stress that often leads to subclinical disease (Jafari et al., 2006). The highest i.v. supply of glutamine (212 g/d) led to lower concentrations of the APP haptoglobin at 7 and 14 d postpartum and greater concentrations of SAA and LPS-binding protein at 7 d relative to controls, without differences in production. Due to its reported function in nonruminants, it was speculated that the increase in SAA with glutamine would help clear endotoxin from the circulation. By the same token, the decrease in haptoglobin was suggested to have been associated with better mucosal barrier function due to glutamine (Jafari et al., 2006).

More recently, formaldehyde-treated glutamine fed at increasing rates (250 g and 350 g/d) from parturition to day 21 postpartum led to greater DMI, milk yield, and plasma glucose, total protein, and albumin without affecting urea-N (Nemati et al., 2018). In addition, glutamine-fed cows had better indices of liver function (lower aspartate aminotransferase) and metabolic stress (lower hydroxybutyare and free fatty acids). The fact that somatic cell counts were markedly lower in those cows suggested a beneficial effect of supplemental glutamine on systemic and mammary gland immune function. Thus, similar to poultry and swine (Chalvon-Demersay et al., 2021), enhanced supply of glutamine might be a practical tool to help cows overcome some of the physiological challenges induced by HS.

Clearly, evaluating mechanistic aspects of AA metabolism during stressful periods in ruminants is more challenging than nonruminants. In addition, concerns for animal welfare have reduced the ability to use surgically prepared or multicatheterized animals for more detailed studies. In that context, the use of plasma concentrations of AA has been proposed as an approach to identify which AA have increased demand during a period of stress (Waggoner et al., 2009; Hoskin et al., 2016). In growing beef steers (~262 kg body weight), the administration of LPS via the jugular vein (2 µg/kg body weight) did not affect DMI, but led to decreases in plasma concentrations of methionine, lysine, leucine, isoleucine, phenylalanine, tryptophan, glycine, serine, and asparagine, while plasma haptoglobin and tumor necrosis-alpha increased 3-fold (Waggoner et al., 2009). Because data from nonruminants indicated that methionine cycle flux and transsulfuration increase during inflammation (Li et al., 2007), e.g., to synthesize GSH, there was interest on whether supplemental rumen-protected methionine (RPMet) during the LPS challenge would help maintain systemic methionine levels. Although RPMet led to greater plasma methionine before LPS, it did not restore concentrations of methionine or other AA after the challenge. To explain the lack of response to exogenous methionine authors suggested that 1) extra metabolizable methionine was either used by the immune system or the liver, 2) methionine might not have been the most-limiting AA, and 3) the decrease in other essential AA confirmed that one or more of these were limiting or co-limiting (Waggoner et al., 2009). Similar type of studies with sheep have allowed a better understanding of the AA required in response to inflammatory stresses (Hoskin et al., 2016).

### Dietary AA supply during heat stress in ruminants

Although few studies are available relative to work in nonruminants, there is recent evidence that altering the AA profile of the diet can improve some features of the HS phenotype. For instance, when compared with diets equivalent in metabolizable protein, increasing dietary proportions of lysine, methionine, and histidine reduced rectal temperature in HS cows (cyclical THI = 76 to 82) (Ruiz-Gonzalez A., 2021). Although dietary AA supplementation did not recover milk or component yields, milk protein concentrations tended to increase. In addition, these changes were accompanied by a reduction in plasma insulin concentration, suggesting modulation of metabolism in response to AA profile. In a subsequent study, the same authors reported increased oxidation rates of AA such as leucine in HS cows relative to pair-fed (Ruiz-Gonzalez, 2022), an effect which was reversed by AA supplementation. These data suggested that dietary AA profile could moderately alter milk composition, impact intermediary metabolism and improve hyperthermia.

Although the work of Pate et al. (2020) and Coleman et al. (2022a, 2022b) discussed briefly in the previous section revealed a number of novel responses at the systemic and tissue level due to HS, at the core of those studies was the use of RPMet (fed at 1.05 g of RPMet/kg of DMI) as a way to mitigate the negative impact of HS on milk production and aspects of inflammation and oxidative stress. Prior to this study, Kassube et al. (2017) attempted to reverse the negative impacts on performance by enhancing the supply of methionine, lysine, and branched-chain AA to HS lactating cows without success. The lack of effect of RPMet on the protein abundance of INSR, SLC2A4, and AKT in the study of Coleman et al. (2022a, b) suggested that enhanced postruminal supply of methionine during HS helped maintain insulin-signaling homeostasis and the gluconeogenic state of the liver. The same cows (fed RPMet under HS) had a lower decrease in milk yield and milk fat compared with the pair-fed thermoneutral controls (Pate et al., 2020).

**Figure 2.**
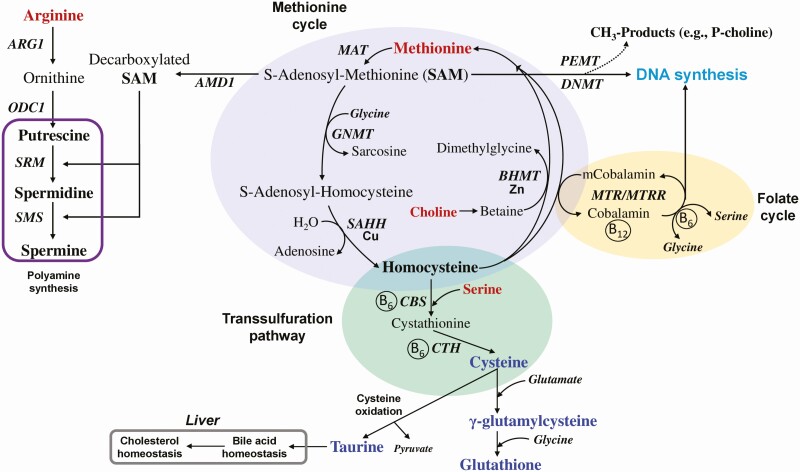
Summary of key reactions in the methionine cycle, folate cycle, and transsulfuration pathway as related to the flow of methionine, choline, S-adenosyl-methionine, the use of several amino acid intermediates, and the synthesis of the antioxidants glutathione and taurine. AMD1, adenosylmethionine decarboxylase 1; ARG1, arginase 1; BHMT, betaine--homocysteine S-methyltransferase; CBS, cystathionine beta-synthase; CTH, cystathionine gamma-lyase; DNMT, DNA methyltransferase 1; GNMT, glycine N-methyltransferase; MAT, methionine adenosyltransferase 1A; MTR, 5-methyltetrahydrofolate-homocysteine methyltransferase; MTRR, 5-methyltetrahydrofolate-homocysteine methyltransferase reductase; ODC1, ornithine decarboxylase 1; PEMT, phosphatidylethanolamine N-methyltransferase; SAHH, adenosylhomocysteinase; SMS, spermine synthase; SRM, spermidine synthase. B_6_, vitamin B_6_; B_12_, vitamin B_12_; Cu, copper; Zn, zinc.

The potential biological synergy between AA and certain trace minerals (TM) in helping ruminants navigate periods of HS is worth mentioning. Dietary AA-TM chelates, which have greater bioavailability than their sulfate counterparts, can alleviate some negative impacts of HS (e.g., gut permemability) in non-ruminants (Sanz Fernandez et al., 2014). Early-lactation cows fed an RPMet-Zinc feed additive during environmental HS in the Summer (average ambient temperature 32.5°C and THI 75.1) for 6-wk not only had lower plasma concentrations of APP and pro-inflammatory cytokines, but also greater antioxidant status, plasma calcium and zinc, energy-corrected milk, milk protein and fat percentage, and DMI (Danesh Mesgaran et al., 2022). Because a mixture of zinc and methionine were fed in this study, the specific effect of each nutrient on the responses to HS could not be discerned with certainty. It is noteworthy, however, that other methionine-containg TM supplements alleviated negative impacts of HS on cow physiology, e.g., selenium-methionine in place of selenium-selenite reduced inflammation and oxidative stress conditions and increased milk yield and DMI (Sun et al., 2019). Mechanistically, it is likely that TM such as zinc and selenium target unique aspects of the response to HS, e.g., selenium is a key cofactor of GSH peroxidase (GPX), and methionine serves as a substrate for the production of antioxidants such as GSH (Figure 2).

### Amino acids, analogs, and one carbon metabolism: role in antioxidant synthesis

One-carbon metabolism encompasses the transfer of carbon atoms in a variety of metabolic reactions and plays a fundamental role in the generation of labile methyl groups (Coleman et al., 2021). Folate, betaine, methionine, and choline, along with several dispensable AA (Figure 2) are key nutrients in this pathway. Considering that methionine is limiting in most diets fed to ruminants and its unique role in one-carbon metabolism, a growing number of studies in the last decade have focused on RPMet supplementation as dietary strategy to “manipulate” various aspects of this pathway, e.g., antioxidant synthesis. Feeding RPMet can increase flux of the transsulfuration pathway, enhancing production of the antioxidants taurine and GSH to help reduce oxidative stress and inflammation during stressful events including HS (Coleman et al., 2020, 2021). Metabolism of N-carbamoylglutamate [NCG, a feed additive used in dairy cows; (Ma et al., 2021)] via carbamoyl-phosphate synthase 1 generates arginine, which can then serve as substrate along with SAM from the methionine cycle to synthesize the polyamines (Figure 2). These molecules are potent antioxidants and when NCG was fed to dairy cows undergoing seasonal HS, it not only reduced plasma cortisol and malondialdehyde (MDA) concentrations, but also it increased total antioxidant capacity of plasma and the activity of SOD and GPX (Ma et al., 2021). Whether choline or betaine, both available as feed additives for cattle, can benefit the antioxidant environment of cattle experiencing HS is largely unknown. Betaine fed to peripartal dairy cows led to greater energy-corrected milk yield (Monteiro et al., 2017), while RP-choline during a short-term HS challenge via heat blankets in late-lactation cows did not alter production or physiological parameters in blood (Holdorf and White, 2021). Additional work during HS with nutrients that feed into one-carbon metabolism appears warranted.

### Hepatic molecular responses in one-carbon metabolism

Data obtained from the study of Coleman et al. (2022a) clearly demonstrated that HS downregulated hepatic expression of *CBS*, *CDO1*, *CTH*, and *GSS* genes, all of which are key regulators of GSH synthesis. Furthermore, activity of methionine adenosyl transferase (MAT) tended to increase with HS, which was taken as an indication of increased flux of the methionine cycle and production of S-adenosyl methionine (SAM). This increase in MAT activity corresponded with a similar increase in cystathionine-β-synthase (CBS) activity, the rate-limiting enzyme in the transsulfuration pathway, suggesting a link between SAM availability and activation of CBS during HS.

The fact that hepatic GSH did not differ due to HS in the study of Coleman et al. (2022a) could be linked to the quick need for antioxidants at the cellular level or needs for taurine (for bile acid synthesis) rather than GSH (Figure 2). It is well established that SOD and GSH are the main antioxidant defense systems in cells and are associated with antioxidant capacity (Coleman et al., 2021). Considering several studies, it is apparent that HS is associated with decreased activity of SOD (Li et al., 2019; Zou et al., 2019) and concentration of GSH (Lu et al., 2019), while MDA (a lipid peroxidation product) increases under periods of heat shock (Li et al., 2019; Zou et al., 2019). In a recent study using HS bovine mammary epithelial cells, it was demonstrated that enhanced supply of taurine restored the activity of SOD while the levels of MDA decreased, all of which indicated that oxidative stress was alleviated (Bai et al., 2021).

### Amino acid supply and the immune response during heat stress

Bovine peripheral blood mononuclear cells exposed to HS experienced a reduction in proliferation (in response to mitogen stimulation), lifespan, and DNA synthesis capacity (Lacetera et al., 2009; Catozzi et al., 2020). Isolated blood polymorphonuclear leukocytes (PMN) had lower phagocytosis and oxidative burst when incubated for 2 h at 41°C compared with 39°C (Lecchi et al., 2016). In addition, in vitro HS-induced PMN lowered the production and release of IL-1β and IL-6 (Lopreiato et al., 2020). This negative effect was corroborated by data on immune and oxidative stress-related genes in the same study. In fact, PMN under HS were characterized by a downregulation of *NFKB1*, *TLR1*, *IL6*, *IRAK1*, *LYZ*, *MPO*, and *SOD1*. The toll-like receptors are pivotal during the innate immune response against pathogens because they recognize foreign nonself material and trigger the innate inflammatory response through activation of nuclear factor (NF)-κB, which in turn leads to upregulation of an array of inflammatory cytokines (Heiser et al., 2018). Hence, expression of an increased number of pattern recognition receptors and their downstream activation of IRAK1 and NF-κB signaling is likely to be beneficial, especially under HS. In fact, their signaling rapidly induces various inflammatory cytokines and chemokines, consequently triggering an array of antimicrobial immune responses (Heiser et al., 2018).

A recent in vitro experiment with blood mononuclear cells from cattle ranked as high, average, and low immune responders based on their cell-mediated immune response and antibody-mediated immune response generated novel data on cell-specific tolerance to HS (Cartwright et al., 2021). For instance, mononuclear cells (lymphocytes and monocytes) from cows identified as having enhanced or high immune response may be more thermotolerant compared with average and low immune responders. This was based on the discovery that cells from high immune responder cattle exhibited enhanced production of HSP70. Furthermore, peripheral blood mononuclear cells (PBMC) from high immune responders had a greater rate of nitric oxide production, another molecule with a role in thermotolerance.

Another important aspect of lymphocyte activity is their proliferation when stimulated by the mitogen ConA. Indeed, Cartwright et al. (2021), reported that PBMC from high responders displayed increased proliferation and enhanced nitric oxide production when stimulated with LPS, following an in vitro HS challenge. Taken together, HS negatively impacts the immune system not only in vivo, where the action of cortisol likely is important (Baumgard and Rhoads, 2013), but also in vitro, suggesting that more complex mechanisms of response to HS exist in immune cells. These may include a lower concentration of glucose entering the TCA cycle to avoid the increase of ROS upon oxidative phosphorylation, misfolded proteins, or energy and AA channeled towards HSP production.

At least in vitro, the mechanisms by which supplemental methionine acts to modulate the immune response and oxidative stress are linked to one-carbon metabolism (Figure 2) and seem to require an adequate level of choline (Coleman et al., 2021). Compared with PMN not supplemented with methionine, HS PMN supplemented with methionine had a greater fold-change in abundance of *TLR2*, *TLR4*, *IRAK1*, *IL1B*, *IL10, NFKB1,* and *MPO.* Clearly, under HS conditions, enhancing the supply of methionine to PMN effectively increases antioxidant production (conferring cellular processes protection from free radicals and ROS), inflammatory signaling, innate immunity, and the cytoprotective characteristics of PMN through upregulation of HSP. Thus, taken together, the modulatory effects of methionine supply on innate immune cells highlight an opportunity to use it during environmental HS.

### Mining Omics data for biomarkers

We used two publicly-available datasets focused on the effect of HS on transcriptome profiles in dairy cow mammary (Gao et al., 2019) and liver (Shahzad et al., 2015) tissue to identify common and unique patterns in expression profiles between datasets. Because of their complementary and functionally-linked metabolic roles, mammary gland and liver are two of the most-important organs helping coordinate lactation in dairy cows. Gao et al. (2019) performed an RNA-sequencing analysis (Illumina HiSeq 4000 platform) on mammary tissue samples collected from cows that were exposed to cyclical HS conditions or remained in thermoneutral conditions. Shahzad et al. (2015) evaluated the effect of Summer and Spring calving season on hepatic molecular adaptations in peripartal cows (*n* = 6 cows in each season biopsied at −30, 3, and 35 days around parturition). Transcriptomic analysis was performed using a 44K Bovine Agilent microarray platform. Both studies uncovered ≥ 2,500 differentially expressed genes (DEG) associated with HS conditions (False discovery rate ≤ 0.05). Although there is interest in combining transcriptome profiles generated from different platforms, the integrative analysis of mixed data between microarray and RNA-sequencing remains challenging. Here we elected to compare the statistically significant results derived from these two studies and described the common and unique functional expression patterns. The complete analyses are available in the Supplemental file.

At a glance, using the 2,437 DEG in mammary tissue for bioinformatics analysis with the Kyoto Encyclopedia of Genes and Genomes (KEGG) pathway database revealed that all but 3 pathways were downregulated (Suppl. Figure S1). The opposite was the case when using the 2,965 DEG from liver tissue of HS cows, where all but 6 pathways were upregulated (Suppl. Figure S2). For the mammary data, these results were not entirely surprising and are in line with the detrimental effects of HS on carbohydrate, amino acid, and lipid metabolism reported in other studies (Tian et al., 2015, 2016). In the context of the liver, besides ‘Energy metabolism’ which had the largest upregulation among metabolic pathways, most of the pathways related to ‘Amino acid metabolism’ and ‘Metabolism of other amino acids’ were upregulated (Suppl. Figure S3). Among those, ‘Cysteine and methionine metabolism’, ‘Histidine metabolism’, ‘Tryptophan metabolism’, and ‘Glutathione metabolism’ were the most impacted and upregulated pathways (Suppl. Figure S3). These results are in general agreement with the reported increases in hepatic use of AA during HS (Baumgard and Rhoads, 2013) or during other stressful conditions (Iseri and Klasing, 2014; McNeil et al., 2016).

When examining a total of 486 DEG shared in mammary and liver of cows undergoing HS, overrepresented KEGG pathways included ‘Steroid biosynthesis’, ‘Fatty acid metabolism’, ‘Propanoate metabolism’, ‘Terpenoid backbone biosynthesis’, and ‘Biosynthesis of amino acids’ pathways (Suppl. Table S5). It is noteworthy that bioinformatics analysis performed on the common DEG suggested a different pattern in terms of up/downregulated pathways, with most of the 7 KEGG metabolic pathways being downregulated in mammary tissue and upregulated in liver tissue (Suppl. Figure S4). The marked upregulation of ‘Metabolism of Other Amino Acids’ along with upregulation of ‘Carbohydrate Metabolism’, ‘Energy metabolism’, and ‘Nucleotide Metabolism’ in liver tissue seemed to highlight the unique function of the liver during HS, i.e., to generate glucose and use AA for the immune and antioxidant response. Unique aspects of the mammary tissue transcriptional response to HS were the upregulation of ‘Cellular processes’ (e.g., ‘Cell Growth and Death’) and ‘Environmental Information Processing’ (e.g., ‘Membrane Transport’). These results resembled closely those of the ruminal epithelium transcriptome in lactating cows experiencing moderate HS and sloughing of the stratum corneum (Guo et al., 2021).

#### Protein–protein interaction network analysis

**Figure 3.**
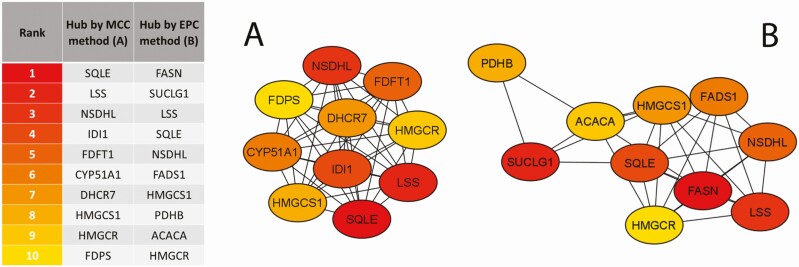
Top-10 ranked hub proteins identified from the protein-protein interaction of the 486 differentially expressed genes that are in common in mammary (Gao et al., 2019) and liver (Shahzad et al., 2015) tissue from cows experiencing heat stress. The networks were developed using the Cytohubba plugin in Cytoscape (Supplementary Material). The hub proteins were uncovered using two algorithms of the cytoHubba plugin, and organized according to (A) maximal clique centrality (MCC) and (B) edge percolated component (EPC). Both of these approaches help predict essential proteins/genes in a biological network, and generate insights into essential regulatory targets. Red to yellow color gradients denote the ranking of hub proteins, from higher to lower according to data in Supplemental Table S8.

The protein-protein interaction (PPI) network generated using STRING (https://string-db.org/) consisted of 486 nodes and 1,481 edges and was built from the interactions among the set of common DEG (i.e., 486). The proteins with several connecting edges can be considered as “hub proteins” and were identified within the PPI network by global and local rank methods developed in the cytoHubba tool (Cytoscape v3.9.1; https://cytoscape.org/) (i.e., edge percolated component, EPC and maximal clique centrality, MCC analyses). Top-10 hub proteins and corresponding interaction network is reported in Figure 3.

Based on shared results between the two approaches, five hub-nodes were identifiable as potential major hub-proteins: SQLE (Squalene Epoxidase), NSDHL (NAD(P) Dependent Steroid Dehydrogenase-Like), LSS (Lanosterol Synthase), HMGCR (3-Hydroxy-3-Methylglutaryl-CoA Reductase), and HMGCS1 (3-Hydroxy-3-Methylglutaryl-CoA Synthase 1) (Figure 3). It is noteworthy that these hubs significantly (False discovery rate *P* ≤ 0.10) enriched ‘Steroid biosynthesis’ and ‘Terpenoid backbone biosynthesis’ pathways, and was consistent with the functional analysis obtained from the 486 common DEG. Such results and a decreased expression of the rate-limiting enzyme for cholesterol synthesis HMGCR, suggested by the downregulation of *HMGCR* gene in the DEG list, supported the hypothesis that this biological process may be downregulated in HS cows as already reported in pigs (Fang et al., 2020). Whether the activity of HMGCR was regulated by cholesterol in a negative feedback manner in order to maintain a relatively stable level of cholesterol is unclear. However, since bile acids are synthesized from taurine and cholesterol in the liver (Figure 2), the scenario of an inhibited cholesterol synthesis is also supported by the downregulation of important genes involved in bile acid synthesis and conjugation: *CYP1A1*, *CYP7A1*, and *CYP27A1*.

Interrogating the ENCODE-Chip-seq database (https://www.encodeproject.org/chip-seq/transcription_factor/) and considering only the common hub-proteins identified, the ChEA3 transcription factor (TF) enrichment analysis (https://maayanlab.cloud/chea3/) revealed three TF as significantly enriched (False discovery rate *P* ≤ 0.10; Suppl. Table S8): SREBF1, SREBF2, and REST. In non-ruminants, SREBF are major regulators of hepatic de novo lipogenesis and esterification controlling the expression of genes involved in the biosynthesis and uptake of fatty acids, triglycerides, cholesterol and phospholipids. The function of REST is mainly as a repressor of transcription. The enrichment of SREBF1 and SREBF2 was compatible with the general scenario depicted in previous sections of the review. Although not yet proven, overall, these data suggested a link between HS and cholesterol synthesis, which could be viewed as an adaptive response to structural membrane damage induced by HS (see previous sections of the review).

## Conclusions

Although the main production outcomes associated with heat stress in ruminants are well-known, physiological impacts beyond the main endocrine and metabolic effects experienced by the animal are just beginning to be understood at the organ level. The vast amount of data in non-ruminants on the role of specific amino acids as “functional nutrients” to help alleviate the inflammatory and oxidative stress state characteristic of the heat-stressed animal offers valuable clues for their application in ruminant production. Combining in vivo research on the application of functional nutrients such as amino acids, potentially in combination with trace minerals, would allow omics tools to verify the biological relevance of the networks identified in the present review, but also help identified novel ones. Targeting dietary amino acid supply to help restore ruminal and intestinal epithelium integrity along with enhancing those amino acids that can feed the one-carbon metabolism pathways to enhance antioxidant synthesis seems achievable in the short-to-medium term. Thus, besides breeding efforts for heat tolerance, combining heat abatement strategies along with enhanced supply of specific amino acids may help maintain health, metabolism, and production during periods of environmental heat stress.

## Supplementary Material

vfad052_suppl_Supplementary_Material

## About the Author

**Juan J. Loor** is a professor in the Department of Animal Sciences and the Division of Nutritional Sciences at the University of Illinois, Urbana-Champaign. He received a BS from the University of California, Davis, and earned an MS and PhD studying lipid nutrition, biohydrogenation, and metabolism in ruminants at Virginia Tech. Since joining the University of Illinois in 2005, his research program has centered on nutritional and physiological genomics during the neonatal period, lactation and rapid growth in beef and dairy cattle. A central goal of his research program is to uncover physiologic regulatory mechanisms induced by nutritional management through integration of ruminal microbiome, circulating metabolome, and tissue transcriptome data.

**Valentino Palombo** is a Postdoctoral Researcher in the Department of Agricultural, Environmental and Food Sciences at University of Molise (Italy). He earned a PhD in Agriculture Technology and Biotechnology with a specialization in Bioinformatics and Data Science. His research interests are mainly towards the application of genomics and transcriptomics in the livestock sector for improving the quality of animal products, but his research activities also include the use of genomic techniques for biodiversity, authentication and traceability purposes.

**Vincenzo Lopreiato** is a research fellow of Animal Sciences in the Department of Veterinary Sciences at the Università degli Studi di Messina, Italy. He received a BS and MS from the Università Cattolica del Sacro Cuore di Piacenza, Italy, and earned a PhD studying nutritional and physiological aspects during the transition period of dairy cows and calves at Università Magna Graecia di Catanzaro, Italy. During his postdoctoral fellowship (2019-2021) at the Università Cattolica del Sacro Cuore di Piacenza he focused on understanding the metabolic and immune adaptations during the transition period of Holstein, Brown Swiss, and Simmental dairy cows. Since joining the Università degli Studi di Messina in 2021, his research program has centered on the impact of agro-industrial waste as feed resource on rumen microbiome, host transcriptome, metabolism, and immune function in beef and dairy cattle dairy cows.

**Mariasilvia D’Andrea** is an associate professor in Animal Breeding and Genetics, Department of Agricultural, Environmental and Food Sciences, University of Molise. She earned a PhD in Biochemistry and applied chemistry and a Master’s Degree in Agricultural Sciences. Her main research focus is on animal breeding, genetic association studies and quantitative genomics in livestock. Her recent research efforts include the identification of chromosomal regions and candidate genes related to novel traits such as composition and quality of animal products by performing studies at the DNA and RNA level, and taking advantage of modern multi-omic approaches for complex traits. These include high-throughput genotyping, whole genome sequencing, and whole genome expression analysis. The goal in applying these techniques are to gain knowledge about biological mechanisms underlying the expression of complex (quantitative) traits, to give insight into the origin and impact of genetic variation, and to explore opportunities for genomics selection strategies.
