# Supplementary material for: Physiological impact of amino acids during heat stress in ruminants
Source: Anim Front. 2023 Oct 13;13(5):69–80. doi: 10.1093/af/vfad052 (PMC10575319; doi:10.1093/af/vfad052)
Supplement: vfad052_suppl_Supplementary_Material [file vfad052_suppl_supplementary_material.docx]

**Supplemental tables and figures**

**Supplemental Table S1.** Dairy cow datasets considered in the bioinformatics analyses.

| **Technology** | **Tissue** | **Total samples** | **References** |
| --- | --- | --- | --- |
| RNA-sequencing/Illumina HiSeq 4000 platform | Mammary gland | 7 samples | Gao ST, Ma L, Zhou Z, Zhou ZK, Baumgard LH, Jiang D, Bionaz M, Bu DP. **Heat stress negatively affects the transcriptome related to overall metabolism and milk protein synthesis in mammary tissue of midlactating dairy cows**. Physiol Genomics. 2019 Aug 1;51(8):400-409. doi: 10.1152/physiolgenomics.00039.2019. Epub 2019 Jul 12. PMID: 31298615. |
| Microarray/Agilent 44K Bovine (V2) | Liver | 12 samples | Shahzad K, Akbar H, Vailati-Riboni M, Basiricò L, Morera P, Rodriguez-Zas SL, Nardone A, Bernabucci U, Loor JJ. **The effect of calving in the summer on the hepatic transcriptome of Holstein cows during the peripartal period**. J Dairy Sci. 2015 Aug;98(8):5401-13. doi: 10.3168/jds.2015-9409. PMID: 26074246. |

**Supplemental Table S2.** Total number of differentially expressed genes (DEGs) retrieved from all datasets are presented against the number of up and downregulated genes in mammary (Gao et al., 2019) and liver (Shahzad et al., 2015) tissue from cows exposed to heat stress. A total of 486 DEG were shared between both studies.

| **Dataset** | **Total DEG  (unique gene with an entrez ID)** | **Upregulated DEG** | **Downregulated DEG** | **DEG identification criteria** |
| --- | --- | --- | --- | --- |
| Gao et al. (2019) | 2,437 | 1,332 | 1,105 | FDR ≤ 0.05 |
| Shahzad et al. (2015) | 2,965 | 1,660 | 1,305 | FDR ≤ 0.05 |

**Supplemental Table S3.** The most enriched KEGG pathways using the list of differentially expressed genes in the mammary tissue of dairy cows experiencing heat stress (Gao et al., 2019) revealed by functional analysis using the online tool DAVID. The functional analysis was performed using the online tool DAVID (https://david.ncifcrf.gov/).

| **KEGG pathway** | **# of Genes** | **%** | **P-Value** | **FDR** |
| --- | --- | --- | --- | --- |
| Steroid biosynthesis | 15 | 0.615510874 | 5.28E-09 | 7.92E-07 |
| Phagosome | 46 | 1.88756668 | 1.34E-06 | 1.34E-04 |
| Central carbon metabolism in cancer | 24 | 0.984817398 | 5.95E-06 | 4.46E-04 |
| Valine. leucine and isoleucine degradation | 20 | 0.820681165 | 7.75E-06 | 4.65E-04 |
| Proteoglycans in cancer | 49 | 2.010668855 | 1.45E-05 | 7.27E-04 |
| Fructose and mannose metabolism | 15 | 0.615510874 | 3.31E-05 | 0.001418625 |
| Propanoate metabolism | 14 | 0.574476816 | 5.03E-05 | 0.001839837 |
| Hypertrophic cardiomyopathy | 27 | 1.107919573 | 5.58E-05 | 0.001839837 |
| Diabetic cardiomyopathy | 49 | 2.010668855 | 6.13E-05 | 0.001839837 |
| Biosynthesis of amino acids | 23 | 0.94378334 | 7.16E-05 | 0.001951887 |
| ECM-receptor interaction | 26 | 1.066885515 | 8.59E-05 | 0.002147548 |
| Carbon metabolism | 30 | 1.231021748 | 1.21E-04 | 0.002800781 |
| Glucagon signaling pathway | 28 | 1.148953632 | 1.65E-04 | 0.003532484 |
| Peroxisome | 24 | 0.984817398 | 2.47E-04 | 0.004628989 |
| PPAR signaling pathway | 24 | 0.984817398 | 2.47E-04 | 0.004628989 |
| Arrhythmogenic right ventricular cardiomyopathy | 22 | 0.902749282 | 3.96E-04 | 0.006834945 |
| Cholesterol metabolism | 17 | 0.697578991 | 4.10E-04 | 0.006834945 |
| Chemical carcinogenesis - reactive oxygen species | 50 | 2.051702913 | 5.02E-04 | 0.007930795 |
| Leishmaniasis | 22 | 0.902749282 | 5.79E-04 | 0.008687043 |
| AMPK signaling pathway | 30 | 1.231021748 | 7.69E-04 | 0.009985488 |
| Staphylococcus aureus infection | 27 | 1.107919573 | 7.71E-04 | 0.009985488 |
| Pyruvate metabolism | 15 | 0.615510874 | 7.95E-04 | 0.009985488 |
| Glycolysis / Gluconeogenesis | 19 | 0.779647107 | 8.02E-04 | 0.009985488 |
| EGFR tyrosine kinase inhibitor resistance | 22 | 0.902749282 | 8.32E-04 | 0.009985488 |
| Thermogenesis | 49 | 2.010668855 | 9.17E-04 | 0.010582333 |
| Dilated cardiomyopathy | 25 | 1.025851457 | 0.001251236 | 0.013902621 |
| Fatty acid metabolism | 17 | 0.697578991 | 0.001920656 | 0.020056513 |
| Oxidative phosphorylation | 32 | 1.313089865 | 0.001938796 | 0.020056513 |
| Glycerophospholipid metabolism | 25 | 1.025851457 | 0.002551636 | 0.025516357 |
| Non-alcoholic fatty liver disease | 35 | 1.436192039 | 0.002649412 | 0.025571719 |
| Complement and coagulation cascades | 23 | 0.94378334 | 0.00272765 | 0.025571719 |
| MAPK signaling pathway | 55 | 2.256873205 | 0.002906632 | 0.025739677 |
| Parathyroid hormone synthesis. secretion and action | 25 | 1.025851457 | 0.002917163 | 0.025739677 |
| Rheumatoid arthritis | 25 | 1.025851457 | 0.003325962 | 0.026934962 |
| Alcoholic liver disease | 33 | 1.354123923 | 0.003354528 | 0.026934962 |
| Malaria | 17 | 0.697578991 | 0.003364542 | 0.026934962 |
| Adipocytokine signaling pathway | 19 | 0.779647107 | 0.003411762 | 0.026934962 |
| Focal adhesion | 40 | 1.641362331 | 0.004602702 | 0.035405401 |
| HIF-1 signaling pathway | 25 | 1.025851457 | 0.005475236 | 0.041064273 |

**Supplemental Table S4.** The most enriched KEGG pathways using the list of differentially expressed genes in the liver of dairy cows experiencing heat stress (Shahzad et al., 2015). The functional analysis was performed using the online tool DAVID (https://david.ncifcrf.gov/).

| **KEGG pathway** | **# of Genes** | **%** | **P-Value** | **FDR** |
| --- | --- | --- | --- | --- |
| Metabolic pathways | 391 | 13.18718381 | 1.06E-23 | 2.99E-21 |
| Lysosome | 51 | 1.720067454 | 3.28E-09 | 4.64E-07 |
| Fatty acid metabolism | 27 | 0.910623946 | 1.35E-07 | 1.28E-05 |
| Ribosome | 53 | 1.787521079 | 3.04E-07 | 2.15E-05 |
| Parkinson disease | 76 | 2.563237774 | 1.39E-06 | 7.89E-05 |
| Carbon metabolism | 38 | 1.281618887 | 3.84E-06 | 1.81E-04 |
| Protein processing in endoplasmic reticulum | 51 | 1.720067454 | 5.80E-06 | 2.34E-04 |
| Chemical carcinogenesis - reactive oxygen species | 64 | 2.15851602 | 1.52E-05 | 5.37E-04 |
| Biosynthesis of amino acids | 27 | 0.910623946 | 2.38E-05 | 7.47E-04 |
| Pathways of neurodegeneration - multiple diseases | 111 | 3.743676223 | 3.61E-05 | 0.00100207 |
| Coronavirus disease - COVID-19 | 71 | 2.39460371 | 3.89E-05 | 0.00100207 |
| Huntington disease | 78 | 2.6306914 | 4.56E-05 | 0.001025615 |
| PPAR signaling pathway | 29 | 0.978077572 | 4.71E-05 | 0.001025615 |
| Salmonella infection | 66 | 2.225969646 | 6.57E-05 | 0.001327572 |
| Thermogenesis | 61 | 2.057335582 | 1.11E-04 | 0.00208531 |
| Cysteine and methionine metabolism | 20 | 0.674536256 | 1.47E-04 | 0.002603565 |
| Endocytosis | 62 | 2.091062395 | 2.01E-04 | 0.003344962 |
| Phagosome | 46 | 1.55143339 | 2.63E-04 | 0.00361793 |
| Oxidative phosphorylation | 40 | 1.349072513 | 2.67E-04 | 0.00361793 |
| Aminoacyl-tRNA biosynthesis | 18 | 0.607082631 | 2.68E-04 | 0.00361793 |
| Arginine and proline metabolism | 19 | 0.640809444 | 2.68E-04 | 0.00361793 |
| Diabetic cardiomyopathy | 54 | 1.821247892 | 3.94E-04 | 0.00484533 |
| Prion disease | 67 | 2.259696459 | 3.99E-04 | 0.00484533 |
| Amyotrophic lateral sclerosis | 86 | 2.900505902 | 4.11E-04 | 0.00484533 |
| Biosynthesis of cofactors | 41 | 1.382799325 | 4.98E-04 | 0.005449016 |
| Regulation of actin cytoskeleton | 54 | 1.821247892 | 5.01E-04 | 0.005449016 |
| Fatty acid degradation | 17 | 0.573355818 | 6.63E-04 | 0.006945529 |
| Autophagy - animal | 39 | 1.3153457 | 7.41E-04 | 0.007083749 |
| Adherens junction | 23 | 0.775716695 | 7.47E-04 | 0.007083749 |
| Ubiquitin mediated proteolysis | 40 | 1.349072513 | 7.51E-04 | 0.007083749 |
| Glycine. serine and threonine metabolism | 17 | 0.573355818 | 8.80E-04 | 0.008037015 |
| Endometrial cancer | 20 | 0.674536256 | 0.001210585 | 0.010706108 |
| Alzheimer disease | 87 | 2.934232715 | 0.001271475 | 0.01090386 |
| Mitophagy - animal | 23 | 0.775716695 | 0.001390441 | 0.011392864 |
| Cholesterol metabolism | 18 | 0.607082631 | 0.001409011 | 0.011392864 |
| Fluid shear stress and atherosclerosis | 38 | 1.281618887 | 0.001463979 | 0.011480821 |
| Ferroptosis | 17 | 0.573355818 | 0.001501026 | 0.011480821 |
| Human T-cell leukemia virus 1 infection | 56 | 1.888701518 | 0.001601531 | 0.011927193 |
| Citrate cycle (TCA cycle) | 13 | 0.438448567 | 0.00165458 | 0.012006314 |
| Complement and coagulation cascades | 27 | 0.910623946 | 0.001816546 | 0.012852064 |
| Peroxisome | 25 | 0.84317032 | 0.001945347 | 0.013427642 |
| AMPK signaling pathway | 33 | 1.112984823 | 0.002446032 | 0.016481599 |
| Histidine metabolism | 10 | 0.337268128 | 0.004058539 | 0.026710849 |
| Human papillomavirus infection | 75 | 2.529510961 | 0.004294471 | 0.027621258 |
| Tryptophan metabolism | 16 | 0.539629005 | 0.005285373 | 0.033239126 |
| Sphingolipid signaling pathway | 31 | 1.045531197 | 0.005412066 | 0.033295972 |
| Propanoate metabolism | 12 | 0.404721754 | 0.005612949 | 0.033797118 |
| Nucleotide metabolism | 24 | 0.809443508 | 0.005904848 | 0.034813998 |
| Hepatocellular carcinoma | 41 | 1.382799325 | 0.007138926 | 0.041230941 |
| Apoptosis | 35 | 1.180438449 | 0.007375218 | 0.041743731 |
| Platinum drug resistance | 22 | 0.741989882 | 0.007656448 | 0.041762323 |
| Legionellosis | 18 | 0.607082631 | 0.007673642 | 0.041762323 |
| Steroid biosynthesis | 9 | 0.303541315 | 0.007828919 | 0.041803472 |

**Supplemental Table S5.** The most enriched KEGG pathways considering 486 differentially expressed genes in common among mammary (Gao et al., 2019) and liver (Shahzad et al., 2015) datasets revealed by functional analysis using the online tool DAVID.

| **KEGG pathway** | **# of Genes** | **%** | **P-Value** | **FDR** |
| --- | --- | --- | --- | --- |
| Metabolic pathways | 111 | 22.83951 | 8.09E-19 | 2.14E-16 |
| Steroid biosynthesis | 9 | 1.851852 | 6.09E-08 | 8.04E-06 |
| Fatty acid metabolism | 11 | 2.263374 | 8.29E-06 | 7.29E-04 |
| Propanoate metabolism | 7 | 1.440329 | 2.91E-04 | 0.01521 |
| Terpenoid backbone biosynthesis | 6 | 1.234568 | 3.43E-04 | 0.01521 |
| Biosynthesis of amino acids | 10 | 2.057613 | 3.46E-04 | 0.01521 |

**Supplemental Table S6.** The most enriched KEGG pathways among the 486 differentially expressed genes that are in common in mammary (Gao et al., 2019) and liver (Shahzad et al., 2015) tissue from cows experiencing heat stress. STRING enrichment analysis considered only the top 10 ranked hub-proteins identified from the protein-protein interaction using the maximal clique centrality (MCC) approach.

| **KEGG pathway** | **# of Genes** | **P-value** | **FDR** | **Genes** |
| --- | --- | --- | --- | --- |
| Steroid biosynthesis | 6 | 4.17E-16 | 1.38E-13 | *CYP51A1, SQLE, NSDHL, FDFT1, DHCR7, LSS* |
| Metabolic pathways | 9 | 4.24E-10 | 7.04E-08 | *CYP51A1, IDI1, SQLE, HMGCR, NSDHL, HMGCS1, FDFT1, DHCR7, LSS* |
| Terpenoid backbone biosynthesis | 3 | 1.38E-07 | 1.53E-05 | *IDI1, HMGCR, HMGCS1* |

**Supplemental Table S7.** The most enriched KEGG pathways among the 486 differentially expressed genes that are in common in mammary (Gao et al., 2019) and liver (Shahzad et al., 2015) tissue from cows experiencing heat stress. STRING enrichment analysis considered only the top 10 ranked hub-proteins identified from the protein-protein interaction using the edge percolated component (EPC) approach.

| **KEGG pathway** | **# of Genes** | **P-value** | **FDR** | **Genes** |
| --- | --- | --- | --- | --- |
| Metabolic pathways | 10 | 3.22E-12 | 1.07E-09 | *SQLE, SUCLG1, HMGCR, NSDHL, HMGCS1, FASN, ACACA, LSS, PDHB, FADS1* |
| Steroid biosynthesis | 3 | 1.38E-07 | 2.29E-05 | *SQLE, NSDHL, LSS* |
| Fatty acid metabolism | 3 | 3.03E-06 | 3.40E-04 | *FASN, ACACA, FADS1* |
| AMPK signaling pathway | 3 | 2.51E-05 | 0.0021 | *HMGCR, FASN, ACACA* |
| Fatty acid biosynthesis | 2 | 3.43E-05 | 0.0023 | *FASN, ACACA* |
| Terpenoid backbone biosynthesis | 2 | 4.70E-05 | 0.0026 | *HMGCR, HMGCS1* |
| Citrate cycle (TCA cycle) | 2 | 1.00E-04 | 0.0049 | *SUCLG1, PDHB* |
| Propanoate metabolism | 2 | 1.10E-04 | 0.0049 | *SUCLG1, ACACA* |
| Pyruvate metabolism | 2 | 1.60E-04 | 0.0058 | *ACACA, PDHB* |
| Glucagon signaling pathway | 2 | 9.80E-04 | 0.0325 | *ACACA, PDHB* |
| Carbon metabolism | 2 | 0.0013 | 0.0395 | *SUCLG1, PDHB* |
| Insulin signaling pathway | 2 | 0.0017 | 0.0474 | *FASN, ACACA* |

**Supplemental Table S8.** Transcription factors (TF) derived from the ChEA3 enrichment analysis (FDR ≤ 0.10), interrogating ENCODE-ChIP-seq and considering five common hub proteins among top 10 ranked hub protein lists obtained from the protein-protein interaction with maximal clique centrality (MCC) and edge percolated component (EPC) approaches.

| **TF** | **Intersect** | **FET**  **p-value** | **FDR** | **Overlapping Genes** |
| --- | --- | --- | --- | --- |
| SREBF1 | 5 | 1.22E-06 | 6.76E-04 | *SQLE. NSDHL. HMGCS1. HMGCR. LSS* |
| SREBF2 | 3 | 3.02E-04 | 0.0793 | *SQLE. HMGCS1. HMGCR* |
| REST | 4 | 4.31E-04 | 0.0793 | *SQLE. HMGCS1. HMGCR. LSS* |

**Supplemental** **Figure S1**. Summary of Dynamic Impact Approach (Bionaz et al., 2012) outcomes considering the main KEGG categories and subcategories using the differentially expressed genes in mammary tissue from cows experiencing heat stress (Gao et al., 2019).

**Supplemental** **Figure S2**. Summary of Dynamic Impact Approach (Bionaz et al., 2012) outcomes considering the main KEGG categories and subcategories using the differentially expressed genes in liver tissue from cows experiencing heat stress (Shahzad et al., 2015).

**Supplemental** **Figure S3**. Summary of Dynamic Impact Approach (Bionaz et al., 2012) outcomes considering ‘Amino acid metabolism’ and ‘Metabolism of Other Amino Acids’ KEGG subcategories using the differentially expressed genes in liver tissue from cows experiencing heat stress (Shahzad et al., 2015).

**Supplementary** **Figure S4**. Summary of Dynamic Impact Approach (Bionaz et al., 2012) outcomes considering the 486 differentially expressed genes in common between the Gao et al. (2019) and Shahzad et al. (2015) datasets.

**REFERENCES**

Bionaz, M., Periasamy, K., Rodriguez-Zas, S.L., Hurley, W.L., Loor, J.J., 2012. A Novel Dynamic Impact Approach (DIA) for Functional Analysis of Time-Course Omics Studies: Validation Using the Bovine Mammary Transcriptome. PLoS One 7 doi:ARTN e32455

10.1371/journal.pone.0032455.
